# Differentiation of *Dmrt1* Z and W Homologs Occurred Independently in Two *Gekko hokouensis* Populations

**DOI:** 10.3390/biom15091293

**Published:** 2025-09-08

**Authors:** Momoka Senga, Nao Kaneko, Yoichi Matsuda, Kazumi Matsubara

**Affiliations:** 1 Graduate School of Bioscience and Biotechnology, Chubu University, 1200 Matsumoto-cho, Kasugai 487-8501, Aichi, Japan; 2 Department of Environmental Biology, College of Bioscience and Biotechnology, Chubu University, 1200 Matsumoto-cho, Kasugai 487-8501, Aichi, Japan; 3 Department of Animal Sciences, Graduate School of Bioagricultural Sciences, Nagoya University, Furo-cho, Chikusa-ku, Nagoya 464-8601, Aichi, Japan

**Keywords:** reptile, sex chromosome, sex determination

## Abstract

*Gekko hokouensis* is a gecko species widely distributed across East Asia. Although most of the Japanese populations possess ZW sex chromosomes (female heterogamety), the degree of sex chromosome differentiation varies among populations. The gene encoding for Dmrt1, a transcription factor involved in testis development in vertebrates, is located on the Z and W sex chromosomes of this species and is therefore a candidate of the sex-determining gene. In this study, we investigated the gene structure of the Z and W homologs of *Dmrt1* in two populations of *Gekko hokouensis* from the Ishigaki Island and Okinawa Island. In the Ishigaki population, the ZW chromosome pair is morphologically undifferentiated, whereas in the Okinawa population the ZW pair is heteromorphic. In the Okinawa population, promoter and exon sequences were nearly identical between the Z and W homologs, and no non-synonymous substitution was detected. In contrast, the W homolog in the Ishigaki population exhibited 42 bp and 12 bp deletions in exon 2. The predicted three-dimensional protein structure revealed a rearrangement of the C-terminal region in the W homolog that may interfere with target site binding. These results indicate that differentiation between Z and W homologs of *Dmrt1* has occurred independently in each population. Our findings highlight the diversity of sex chromosome evolution and sex-determining mechanisms even within a single species.

## 1. Introduction 

Sex-determining systems are highly diverse across reptile lineages [1,2,3,4]. Three primary systems of sex determination are genotypic sex determination (GSD) with male heterogamety (XX/XY) or female heterogamety (ZZ/ZW), both including systems with multiple sex chromosomes, and temperature-dependent sex determination (TSD). These systems are distributed in a seemingly haphazard fashion across the squamate phylogeny [3,5,6], suggesting that transitions between sex determination modes have occurred frequently during evolution [4,7]. Consequently, novel sex chromosomes must have arisen multiple times in these lineages. Gekkonidae is one of the squamate family in which many transitions of sex chromosomes have occurred [8]. Thus, geckos are an excellent animal model to investigate the turnover of sex determination systems and sex chromosome evolution.

**Figure 1:**
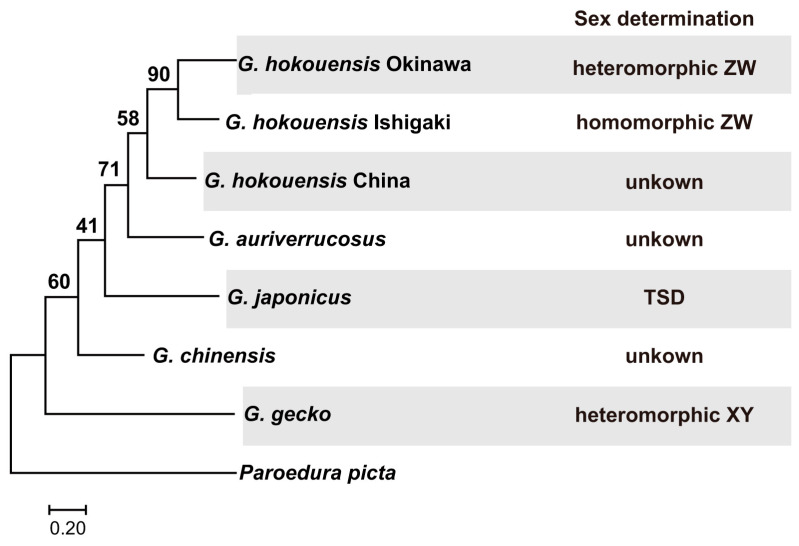
Molecular phylogenetic tree of gecko *COI* genes and sex determination systems. A maximum-likelihood tree was constructed with a 658 bp alignment of *COI* genes from six gecko species including five *Gekko* species distributed in East Asia. *Paroedura picta* was used for the outgroup. Bootstrap values are shown for each node. The sex determination systems of *Gekko* species are shown on the right of the phylogenetic tree.

The genus *Gekko* includes species with TSD, species with XY sex chromosome pairs, and species with ZW sex chromosome pairs (Figure 1). For instance, Schlegel’s Japanese gecko (*G. japonicus*) exhibits TSD [9,10] while the tokay gecko (*G. gecko*) has large XY sex chromosomes [11]. The Kwangsi gecko (*G. hokouensis*) is widely distributed across southeastern China, Taiwan, most islands of the Ryukyu Archipelago, and southern Kyushu, Japan [12]. All populations possess a diploid chromosome number of 2*n* = 38 [12,13]. One continental population of this species lacks heteromorphic chromosomes [12] leaving its mode of sex determination unresolved. Similarly, individuals from the Yaeyama Islands (e.g., Iriomote and Kuroshima Islands) show no apparent heteromorphic chromosome pair [12]. However, G-banding of individuals from the Iriomote Island revealed a different banding pattern on the fourth chromosome pair in females, suggesting that this pair represents a morphologically subtle ZW sex chromosome system [12]. In contrast, conspecific geckos from the Okinawa Island and several other islands of the central and northern Ryukyu Archipelago exhibit a clearly heteromorphic ZW sex chromosome pair [12,13]. These observations suggest that sex chromosome differentiation occurred stepwise during the geographic expansion of the species across the Ryukyu Archipelago.

The sex chromosomes of *G. hokouensis* are particularly interesting because the ZW pair of Okinawa population is homologous to the avian ZW sex chromosomes [13]. In birds, the master sex-determining gene is *Dmrt1* [14], one of the most common testes-determining gene in vertebrates [15]. In medaka fish, a duplicate copy of this gene on the Y chromosome, *Dmy*, plays a role in male determination [16,17]. In the frog *Xenopus laevis*, a W-specific copy of this gene, *Dm-w*, exerts a dominant negative effect on the transcriptional activities of autosomal *Dmrt1*, thereby blocking testis development in ZW embryos [18,19]. Since these discoveries, *Dmrt1* has been implicated in sex determination across a range of vertebrates with both GSD and TSD systems [15]. For a TSD example, epigenetic regulation in turtles results in higher *Dmrt1* expression during the thermosensitive period in embryos incubated at male-producing temperatures compared to those at female-producing temperatures [20,21,22].

In birds, sex determination is thought to be driven by a dosage effect of *Dmrt1*, with males carrying two copies and females one copy of the gene [14,23]. Although *Dmrt1* is also a candidate of the sex-determining gene in *G. hokouensis*, it differs from birds in that the gene has alleles on both of the Z and W chromosomes [13]. If the gene plays a role at the top of sexual differentiation cascade in *G. hokouensis*, the W-linked homolog would be expected to have critical differences from the Z-linked homolog, such as in regulatory regions or coding sequence, that could affect gene expression or function.

In this study, we identified the genomic region containing the *Dmrt1* gene in the draft genome assembly of *G. hokouensis* and characterized and predicted the coding and promoter sequences of the Z and W homologs from Ishigaki Island and Okinawa Island populations. Sequence comparisons revealed independent differentiation of the Z and W homologs between the two populations. We also conducted cytogenetic analyses for the Ishigaki Island populations to characterize their karyotypes. Finally, we discuss potential differences in sex determination mechanisms between these geographically separated populations.

## 2. Materials and Methods

### 2.1. Animals

Five male and four female *Gekko hokouensis* were captured at Arakawa, located in the southern region of Ishigaki Island in the Ryukyu Archipelago, and subsequently maintained and bred in our laboratory. Tail tips were collected from three males and three females for DNA extraction and cell culture. All procedures related to animal collection, handling, sampling, and care were conducted in accordance with the guidelines of Okinawa Prefecture and were approved by the Animal Experiment Committee of Chubu University (Approval Nos. 202010011 and 202310003). For RNA extraction, we used frozen tissue samples from an adult male and an adult female *G. hokouensis* originally collected in Nakagusuku, in the southern region of Okinawa Island (Ryukyu Archipelago, Japan).

### 2.2. Phylogenetic Analyses for Gekko COI Gene

Genomic DNA was extracted from three males and three females of Ishigaki population using the DNeasy Blood and Tissue Kits (Qiagen, Venlo, The Netherlands) following the manufacturer’s protocols. DNA of three males and three females from the Okinawa population were obtained from existing laboratory collections. Approximately 700 bp of the mitochondrial cytochrome oxidase subunit I (*COI*) gene were amplified by PCR using Takara Ex Premier polymerase (Takara) and the universal COI primer pair LCO1490 and HCO2198. Amplicons were sequenced using an ABI 3500 Genetic Analyzer (Thermo Fisher Scientific, Waltham, MA, USA) following sequencing reactions performed with the BigDye Terminator v3.1 Cycle Sequencing Kit (Thermo Fisher Scientific) according to the manufacturer’s instructions. The resulting sequences have been deposited in the International Nucleotide Sequence Database (INSD) under GenBank accession numbers LC885256-LC885269.

To examine the relationships among the populations of *Gekko hokouensis*, we performed phylogenetic analysis based on mitochondrial *COI* sequences. *COI* sequences of other *Gekko* species distributed in East Asia were obtained from INSD: *G. auriverrucosus* (EU417716), *G. chinensis* (KP666135), *G. gecko* (AY282753), *G. japonicus* (KT005800) and *G. hokouensis* Chinese population (KT005801). Phylogenetic relationships were inferred using MEGA11 [24]. *COI* sequences from another gekkonid, *Paroedura picta* (KR149293), were used as outgroups. The best-fitting model for maximum-likelihood (ML) tree construction was selected based on the Bayesian information criterion (BIC) in MEGA11. Tree robustness was assessed by bootstrap resampling with 1000 random replicates.

### 2.3. Chromosome Preparation

Metaphase chromosome spreads were prepared from fibroblast cell lines derived from tail tissues. Briefly, minced tail tissues were implanted in 100 mm culture dishes containing 199 medium (Thermo Fisher Scientific) supplemented with Fetal Bovine Serum (FBS) (Thermo Fisher Scientific) and allowed to propagate under conditions of 26 °C and 5% CO_2_. Once the fibroblast cells reached approximately 80% confluency, they were subcultured into 100 mm culture dishes and passaged up to six times before the chromosomes were harvested. Colcemid (Nacalai, Kyoto, Japan) was added to the culture dish at a final concentration of 125 ng/mL approximately 3 h before harvesting. Harvested cells were then incubated in 0.075 M KCl for 20 min and fixed in 3:1 methanol:acetic acid solution and dropped onto glass slides. The slides were air-dried and stored at −80 °C until further use.

### 2.4. Karyotyping and C-Banding

Conventional Giemsa staining and C-banding were performed on chromosome slides from Ishigaki geckos. C-banded chromosomes were obtained using the CBG (C-bands by Barium hydroxide using Giemsa) method [25] with slight modification. Slides were first treated with 0.2 N HCl for 40 min and rinsed with distilled water. They were then denatured in 5% Ba(OH)_2_ at 50 °C for 5 min. Denaturation was halted by rinsing the slides again with 0.2 N HCl followed by distilled water. The slides were renatured by incubation in 2 × SSC at 60 °C for 60 min, rinsed with distilled water and stained with 4% Giemsa for 30 min.

### 2.5. De Novo Genome Sequencing and Identification of Contigs Containing Dmrt1 Genome Sequences

Lung tissue from a female captured on Okinawa Island was transported to Macrogen, Japan where DNA was extracted and high-throughput sequencing was performed using the PacBio Sequel II System. *De novo* genome assembly was performed using wtdbg2 (v2.3). For error correction, reads were mapped back to the assembled contigs using Arrow and a higher-quality consensus sequence was generated.

To identify the contig containing the *Dmrt1* gene, we performed a BLAST (v2.12) search using the cDNA sequence of *G. hokouensis Dmrt1* (GenBank accession no. AB326222) as the query. This search identified contig 1556. If the sequences Z and W chromosomes were highly diverged, we would expect two contigs sharing similarities to be recovered. We thus also conducted a BLAST search using the sequence of contig 1556 as a query against the draft genome *G. hokouensis* to identify any additional homologous contigs. Furthermore, we searched public DNA databases using contig 1556 as a query to identify additional genes located on the same contig.

Protein-coding sequences of *Dmrt1* within contig 1556 were predicted using the Genewise program available through the EMBL-EBI website [26], with reference amino acid sequences from human (NP_068770), chicken (XP_040511578), and leopard gecko (XP_054842930). Based on these predictions, we annotated the coding sequence of *Dmrt1* in contig 1556. To validate the predicted coding sequence, we cloned the cDNA containing the predicted full-length coding sequence of a homolog of *Dmrt1* in *G. hokouensis*. Total RNA was extracted from testicular tissue of an adult male of Okinawa population using Isogen II (Nippon Gene, Tokyo, Japan). The cDNA was synthesized by RT-PCR using ReverTra Ace (Toyobo, Osaka, Japan), and was used as the PCR template to amplify the homolog of *Dmrt1*. Approximately 1.1 kb cDNA fragments containing the full-length protein coding sequences of *Dmrt1* gene were amplified by PCR using the Takara Ex Premier polymerase (Takara, Kusatsu, Japan) against a testis transcriptome. The sequences of primer pairs are shown in Table 1. The PCR conditions were as follows: an initial denaturation at 94 °C for 1 min, followed by 30 cycles of 98 °C for 5 s, 60 °C for 15 s and 68 °C for 15 s; and finally 68 °C for 5 min for a final extension. The PCR products were cloned using the TOPO blunt-end Cloning Kit (Thermo Fisher Scientific). We sequenced four cDNA clones by using an ABI 3500 Genetic Analyzer (Thermo Fisher Scientific) following a sequencing reaction with BigDye Terminator v3.1 Cycle Sequencing Kit (Thermo Fisher Scientific) and universal primers, M13-F or -R.

### 2.6. Comparison of Gene Structure of Dmrt1 Z and W Homologs in Two G. hokouensis Populations

We designed PCR primers to amplify the predicted promoter region and exons 1 to 5 of the *Dmrt1* gene (Table 1). PCR and sequencing were conducted according to the protocol described in the previous section. Genomic DNA from three males and three females from each population (Okinawa and Ishigaki) was were used as a PCR template. A female-specific sequence was identified as a part of the genome sequence of the *Dmrt1* W homolog.

To predict promoter regions, we search for consensus sequences of binding sites of general transcription initiation factors, including TATAWAW (TATA box), SSRCGCC (BRE: TFIIB recognition element), RTDKKKK (BREd), YYANWYV (Inr: initiator element), RGWYV (DPE: downstream promoter element), CSARCSSAAC (MTE: motif ten element), CTTC–CTGT-AGC (DCE: downstream core element), CCAAT (CAAT box), GGGCGG (GC box).

The predicted protein structure of the *Dmrt1* Z and W homologs of *G. hokouensis* was analyzed using AlphaFold2 [27]. Amino acid sequences of human *DMRT1* (NP_068770) and leopard gecko *Dmrt1* (XP_054842930) were also analyzed.

## 3. Result

### 3.1. Phylogeney of Gekko *COI* Gene

Maximum-likelihood trees were constructed using the Tamura-Nei model with a discrete Gamma distribution for a 658 bp alignment of *COI* gene sequences from six gecko species. In the phylogenetic tree, *G. gecko* was the first to diverge from the other *Gekko* species (Figure 1). *Gekko auriverrucosus* formed a sister group to the *G. hokouensis* clade. Within the *G. hokouensis* clade, the Chinese population diverged first from the two Japanese populations. This pattern of divergence appears to reflect the geographic relationships among the three populations. The two Japanese populations formed a monophyletic group, suggesting that they share a common ancestor. We were unable to infer the ancestral state of the sex determination system for the five *Gekko* species; however, at least two turnovers of the sex determination system have occurred during speciation of the five species.

### 3.2. Karyotype and C-Banding in the Ishigaki Population

**Figure 2:**
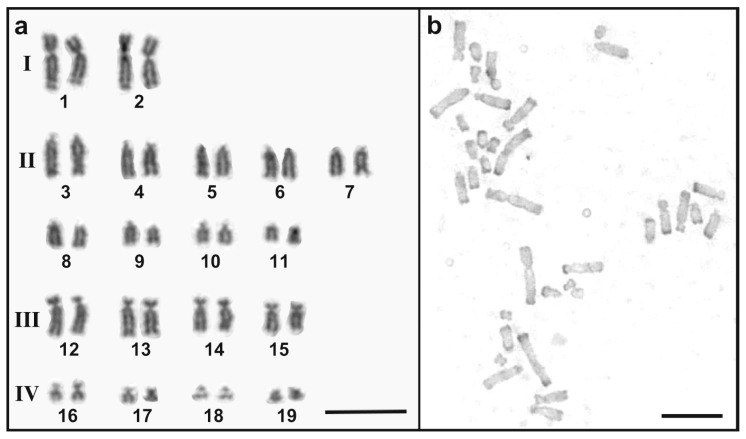
Karyotype and C-banded chromosomes of a *G. hokouensis* from Ishigaki population. (**a**) Giemsa-stained chromosomes of a metaphase of a female individual of *G. hokouensis* Ishigaki population were aligned according to a previous study [12]. Roman numerals show the grouping of chromosomes (see text). Arabic numerals show chromosome numbers. The fourth pair is the provisional ZW sex chromosome pair [12]. (**b**) C-banded metaphase spreads of the same individual. Scale bars indicate 10 µm.

All individuals from the Ishigaki population had karyotypes with 38 chromosome pairs (Figure 2a). The karyotype consists of two pairs of large metacentric chromosomes (group I in Figure 2a), nine pairs of telocentric chromosomes (group II), four pairs of subtelocentric chromosomes (group III), and four pairs of small meta or submetacentric chromosomes (group IV). No notable intra-population variation in chromosome morphology was observed and no heteromorphic chromosome pair was detected in either males or females. The karyotypes examined here are consistent with those previously reported for the *G. hokouensis* population on Iriomote Island [12]. The fourth pair of chromosomes, a telocentric pair, represents provisional Z and W chromosomes in female individuals of Ishigaki Island population (Figure 2a).

C-banding revealed heterochromatin at the telomeres of nearly all chromosomes (Figure 2b). No prominent centromeric heterochromatin was observed and no interstitial C-bands were detected between the centromeric and telomeric regions on any chromosome. This C-banding pattern is consistent with that of the Okinawa population, except for the W-chromosome. In the Okinawa population, the submetacentric W chromosome is marked by a large block of centromeric heterochromatin [12].

### 3.3. Identification of Genome Sequences Containing *Dmrt1* Gene

We obtained a draft genome sequence of a female *Gekko hokouensis* from Okinawa Island. The draft genome consisted of 4293 contigs with a total length of 2,648,808,985 bp. The contig N50 was 2,830,446. The maximum, minimum and average contig lengths were 17,880,836 bp, 3231 bp, and 617,006 bp, respectively.

**Figure 3:**
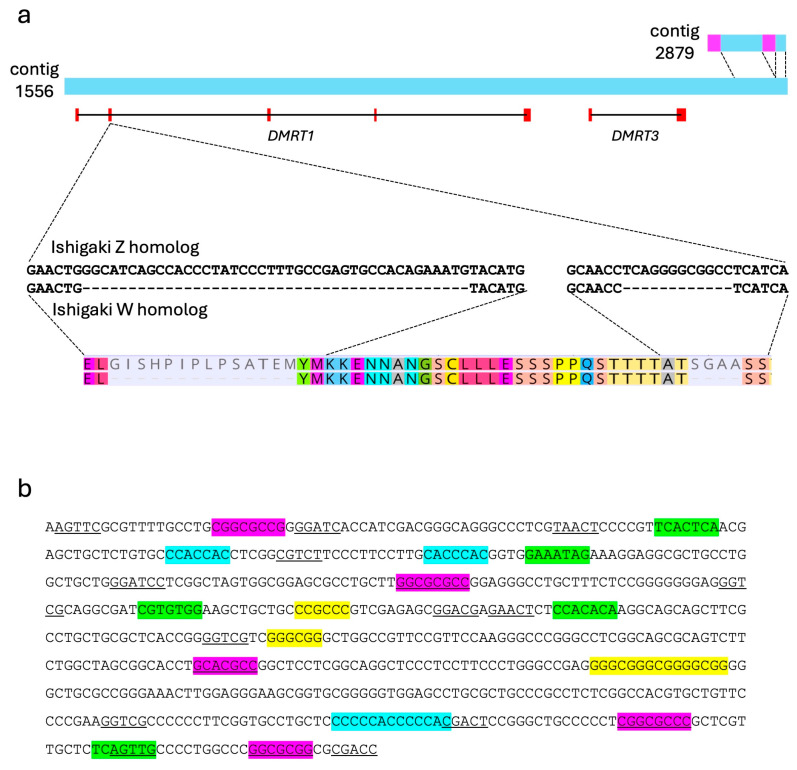
Schematic representation of *Dmrt1*/*Dmrt3*-containing contigs and regulatory sequence features. (**a**) Gene locations of *Dmrt1* and *Dmrt3* in contig 1556. Contig 2879 shares sequence homology with a downstream region of contig 1556 (light blue) and includes two regions containing specific sequences (pink). Partial nucleotide sequences of exon 2 of the Ishigaki Z and W homologs, and corresponding amino acid sequences are shown under the scheme of contigs. Two deletions were detected in exon 2 of the Ishigaki W homolog. (**b**) Predicted sequence of *Dmrt1* promoter region. Consensus binding motifs for general transcription initiation factors are highlighted; purple for BRE, green for BREd, blue for Inr, yellow for GC box (GGGCGG), and underline for DPE (RGWYV).

Using a BLAST search with the *G. hokouensis Dmrt1* cDNA sequence as a query, we identified contig 1556 (accession number BAAIAJ010001556) as containing the genome sequence of *Dmrt1*. This contig was 133,825 bp in length. We also identified another contig, contig 2879 (accession number BAAIAJ010002879) with a length of 14,575 bp, which showed high sequence similarity to the downstream region of contig 1556 (Figure 3a).

Five coding regions of *Dmrt1* were predicted on contig 1556 using ***GeneWise***. This gene structure, comprising five exons, is consistent with that of most vertebrate *Dmrt1* homologs, where the start and stop codons are located in the first and fifth exons, respectively. The *Dmrt1* spans from approximately position 2500 to 88,500 in contig 1556. Additionally, the other DM domain gene, *DMRT3*, was identified on the distal region of this contig (Figure 3a). Thus, contig 2879 shared sequence similarities with contig 1556 on the downstream region of *DMRT3* (Figure 3a). Two unique regions were identified within contig 2879. We verified female-specificity of one of the two regions in Okinawa population using PCR (Supplementary File S1). Primer pairs were designed to span the boundaries between shared and unique regions—one targeting contig 1556 and the other the boundary region (Supplementary File S1). The amplicon from contig 1556 was detected in all individuals from both the Okinawa and Ishigaki populations. In contrast, the amplicon from the boundary region was detected only in females from the Okinawa population (Supplementary Figure S1b). These results suggest that contigs 1556 and 2879 correspond to genomic sequences from the Z and W chromosomes, respectively, of Okinawa population. Furthermore, the female-specific region of contig 2879 was not detected in genomic DNA from females of the Ishigaki population, indicating that this region may be unique to the Okinawa W chromosome.

We cloned cDNA which contains the full-length coding sequence of *Dmrt1* (accession no. LC885331) and the sequence was matched to predicted coding sequences. Although the transcription start site and transcription termination site could not be identified in this study, we inferred their approximate positions by comparison with the cDNA sequence of *G. japonicus* (accession no. PV253953). Based on this comparison, the transcription start site (TSS) was estimated to be located approximately 200 bp upstream of the start codon, and the transcription termination site (TTS) approximately 1000 bp downstream of the stop codon.

### 3.4. Gene Structure of G. hokouensis Dmrt1

We sequenced the predicted promoters, exon 1 through to 5 inclusive of the Z and W *Dmrt1* homologs in Okinawa and Ishigaki populations (accession numbers LC885400–LC885402, LC885407–LC885410). The nucleotide sequences of all exons of Z homolog were highly conserved between the two populations. Between the Okinawa and Ishigaki Z homologs, we identified two synonymous substitutions in the coding region of exon 1, one single nucleotide polymorphism (SNP) in the 3′ untranslated region (UTR), and two SNPs in the 5′ UTR. Comparison of the Z and W homologs in the Okinawa population revealed two SNPs in the 5′ UTR, but no non-synonymous substitutions in the coding region. Consequently, the Z and W homologs from Okinawa, as well as the Z homolog from Ishigaki, encode identical amino acid sequences. In contrast, the W homolog from the Ishigaki population exhibited one synonymous substitution in exon 2 and a single nucleotide polymorphism (SNP) in the 5′ UTR. In addition, two deletions of 42 bp and 12 bp were detected in exon 2 (Figure 3a). No male specific sequence was detected in any exons in the two populations. These results support the presence of female heterogametic sex chromosomes (ZW system) in both populations. This conclusion holds despite the absence of a visibly heteromorphic chromosome pair in karyotypes of individuals from the Ishigaki Island population.

Although several interspecific variations, such as SNPs and copy number variations in repeat units, were detected in the predicted promoter region, no sex specificity was detected. Consensus binding motifs for general transcription initiation factors were detected within 650 bp upstream from the start codon. These included five sites matching the bRE motif (SSRCGCC), five BREd sites (RTDKKKK), three Inr sites (YYANWYV), 12 DPE sites (RGWYV) and four GC box motifs (GGGCGG).

### 3.5. Predicted Protein Structure of Dmrt1 Z and W Homologs

**Figure 4:**
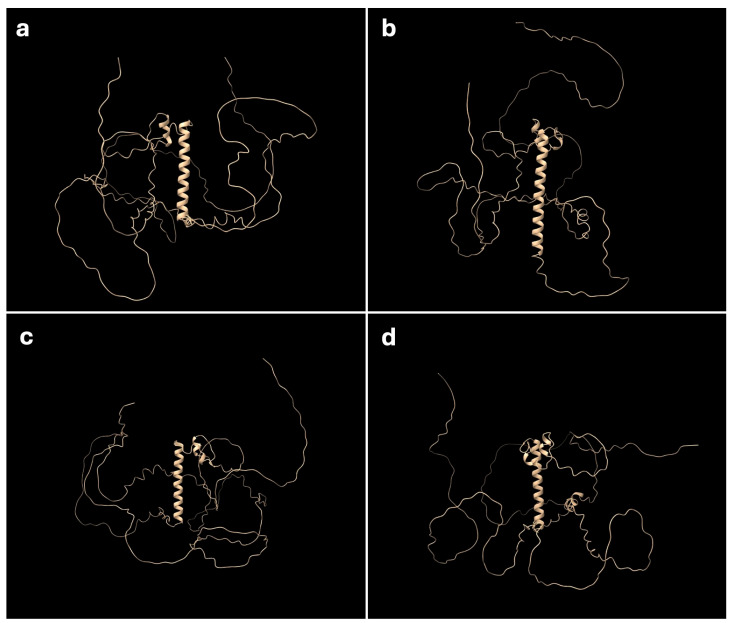
Predicted 3D protein structure of DMRT1. Three-dimensional protein structure predicted using AlphaFold2. (**a**) *Dmrt1* Z homolog of *G. hokouensis* Ishigaki population, (**b**) the W homolog, (**c**) human *DMRT1*, (**d**) leopard gecko *Dmrt1*. Amino acid sequence translated from exon 2 forms an α-helix structure, which is located on the central position of the protein.

To investigate the functional differences between the Z and W *Dmrt1* homologs in the Ishigaki population, we predicted their protein structures using AlphaFold2. As the Okinawa Z and W homologs share identical amino acid sequences with the Ishigaki Z homolog, they were excluded from this analysis. Human and leopard gecko homologs were included as references. In all four homologs, a single central α-helix was predicted (Figure 4a–d). This helix corresponds to the DM domain, which spans exons 1 and 2, and is thought to bind the major groove of target DNA sequences to activate transcription of downstream target genes. Although the Ishigaki W homolog lacks 18 amino acid residues in total, these deletions did not affect the formation of the α-helix. However, a notable structural difference was observed at the C-terminal end of the Ishigaki W homolog, which appears to fold over one side of the protein structure (Figure 4b). This arrangement was not observed for the human, leopard gecko and the Ishigaki Z homologs (Figure 4a,c,d).

## 4. Discussion

In this study, we determined the genome sequence of *Dmrt1* Z and W homologs in two *Gekko hokouensis* populations—those from Ishigaki Island and Okinawa Island—and compared their gene structures within and between populations. Although the differentiation status of Z and W chromosomes differs between the two populations, both populations have female heterogametic sex chromosomes (ZW system) [12,13]. This initially suggested that the early stages of sex chromosome differentiation, and potentially the underlying sex determination mechanism, might be shared between the populations. However, the *Dmrt1* W homolog sequences were different between the two populations, whereas the Z homolog sequences were almost identical. This finding suggests that differentiation of the *Dmrt1* Z and W occurred independently in each population. Furthermore, a W-specific sequence located downstream of *Dmrt3* was identified only in Okinawa population, providing additional evidence for independent differentiation. Intraspecific variation of sex chromosomes and the gametologous *Dmrt1* genes was reported in a European *Rana* frog [28]. In this frog, the degree of differentiation of sex chromosomes and *Dmrt1* is associated with the phylogeography history of this species. Similarly, in *G. hokouensis,* it is plausible that differentiation of sex chromosomes occurred after dispersal of this species throughout the Ryukyu Archipelago.

The predicted promoter sequences were identical between the Z and W homologs in both populations. This suggests that, in the absence of epigenetic differences, general transcription factors could potentially activate transcription from both homologs. In *Xenopus laevis* and *Silurana tropicalis*, *Dmrt1* expression is regulated by two distinct promoters—one for regulation of the expression in germ cells and another for somatic cells [29]. Therefore, analysis of a longer upstream region beyond the proximal promoter is necessary to clarify the regulatory mechanism in geckos.

The Ishigaki W homolog lacks two segments—42 bp and 12 bp—in exon 2. Notably, a partial sequence duplication from exon 2 to 3 of *Dmrt1* was found in an SRY-negative human boy with a 46,XX chromosomal complement and a disorder of sexual development [30]. Sex reversal by deletion of exon 2 of *Dmrt1* has not been reported in vertebrates so far. Although the two deletions on exon 2 in Ishigaki W homolog did not affect the formation of the α-helix (an essential DNA binding structure) in the predicted 3D model, the C terminal of protein seems to fold over and partially obscure the protein’s binding surface. This altered conformation may impair binding affinity to target DNA sites and potentially influence the protein’s regulatory function.

The mechanism of sex determination in the Okinawa population remains unclear based on current data, as the Z and W homologs of *Dmrt1* have almost identical sequences in both the predicted promoter regions and exons. However, we did identify two W-specific parts in the distal region of *Dmrt3* in the Okinawa population. Similar to *Dmrt1*, *Dmrt3* is a transcription factor containing a DM domain and has been implicated in testicular development. Although knockout of *Dmrt3* in laboratory mice does not affect testis development [31], missense mutations in *Dmrt3* and *OAS3* have been associated with disorders of sexual development in 46,XY individuals in humans [32]. Thus, we need to compare the gene structure of the Z and W homologs of *Dmrt3* in the future. Chromosome morphology also differs between W chromosomes of Okinawa and Ishigaki populations. The W chromosome in the Okinawa population is subtelocentric and has a prominent block of centromeric heterochromatin [12]. Notably, *Dmrt1* is located within the centromeric region of the W chromosome [13], suggesting that its expression may be suppressed by the surrounding heterochromatin, potentially through a mechanism similar to position-effect variegation. These observations highlight the need to investigate whether transcriptional repression of the W-linked *Dmrt1* occurs through epigenetic mechanisms such as chromatin remodeling.

## 5. Conclusions

In this study, we showed the independent differentiation of gametologous *Dmrt1* genes between two populations of *Gekko hokouensis*. This finding suggests that two conspecific populations have different sex determination mechanisms. However, further research—including genomic and functional analyses—is needed to fully elucidate these mechanisms and to understand the evolutionary processes underlying sex chromosome differentiation and the role of sex determination genes. These results further underscore the remarkable diversity of sex chromosome systems and master sex-determining factors among vertebrates. *Gekko hokouensis* represents a particularly promising model for such research, given its wide geographic distribution and the discovery of multiple genetically distinct conspecific strains.

## Figures and Tables

**Table 1:** Primer sequences used for amplification of the *Dmrt1* coding region, individual exons, and promoter region in *Gekko hokouensis*. Forward (Fw) and reverse (Rv) primers are shown in the 5′–3′ orientation for each targeted region.

Target		Primer Sequence (5′–3′)
*DMRT1* coding sequence	Fw	CCTCCTTCAGCAAGCCCTCGG
	Rv	CAGTGAATGTGCCAGAATCGGAGGT
*DMRT1* exon 1	Fw	CGGGAAACTTGGAGGGAAGCGG
	Rv	CCCCGTCGATCCTTTGCAAGCC
*DMRT1* exon 2	Fw	AGGTTTTGTGCAGCTGTTTTGTGCA
	Rv	ATAAAGGCCTTACAACCCTGCACCC
*DMRT1* exon 3	Fw	AGTCACTGGGAATGCTTTGGTCCTG
	Rv	GGAGTCATTCCGTCAATGCCAAAGC
*DMRT1* exon 4	Fw	CAGCACAAAGATTCTTAAGTAGGCA
	Rv	GACTGGATGACTTCTGCCTAGTATT
*DMRT1* exon 5	Fw	CCCCTTTCTCCATTGCCTCCTCAG
	Rv	CTTGAATGGACAGCACTGAAACGGC
*DMRT1* promoter	Fw	ATCGGGTTTTTCGTTCTGCCCAGAA
	Rv	GACCTTCGGGGAACAGCACGTG

## Data Availability

The nucleotide sequence data generated in this study are available from the INSD. The accession numbers are LC885256–LC885269 for the *COI*, and BAAIAJ010001556 and BAAIAJ010002879 for the two contigs in the *G. hokouensis* draft genome, LC885331 for the cDNA sequences of *Dmrt1 Z* homolog, and LC885400–LC885402 and LC885407–LC885410 for the genomic DNA sequences of *Dmrt1 Z* and W homologs.

## Supplementary Materials

The following supporting information can be downloaded at: https://www.mdpi.com/article/10.3390/biom15091293/s1. File S1:  Identification of a contig derived from the W chromosome in Okinawa population, and test for conservation of a W specific sequence between the two populations; Figure S1: Schematic diagram of primer positions on the contigs (a) and gel electrophoresis image for PCR amplicons (b).
