# Supplementary material for: Differentiation of Dmrt1 Z and W Homologs Occurred Independently in Two Gekko hokouensis Populations
Source: Biomolecules. 2025 Sep 8;15(9):1293. doi: 10.3390/biom15091293 (PMC12467647; doi:10.3390/biom15091293)
Supplement: Supplementary file 1 [file biomolecules-15-01293-s001.zip › biomolecules-3737801-Figure_S1_revised_3rd.pdf]

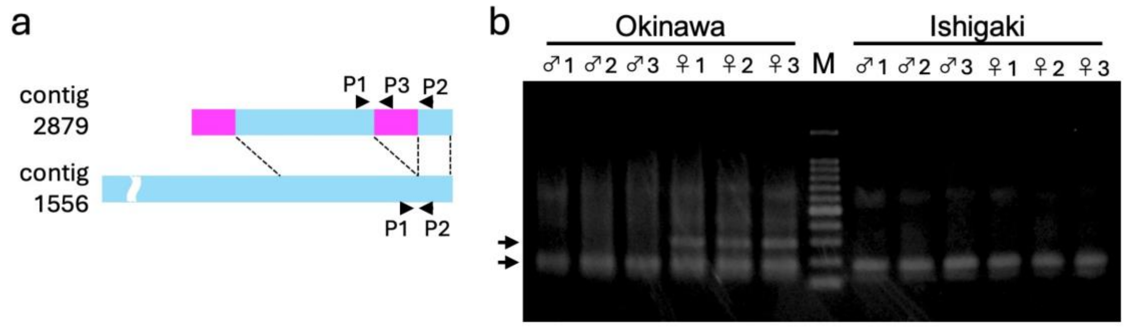

Supplementary Figure S1. Schematic diagram of primer positions on the contigs (a) and gel electrophoresis image for PCR amplicons (b). (a) Arrow heads indicate the relative positions of primers, P1: Gho_ZW_Fw, P2: Gho_ZW_Rv, P3: Gho_W_Rv. The light blue parts indicate the common sequence between the two contigs and the pink parts indicate the specific sequence in contig 2879. (b) Electrophoresis with agarose gel for the PCR amplicons. Arrows indicate the bands by the amplicons. The sizes of small and large amplicons were estimated at c.a. 180 bp and c.a. 280. M: 100 bp ladder DNA marker (Takara).
