# Supplementary material for: Differentiation of Dmrt1 Z and W Homologs Occurred Independently in Two Gekko hokouensis Populations
Source: Biomolecules. 2025 Sep 8;15(9):1293. doi: 10.3390/biom15091293 (PMC12467647; doi:10.3390/biom15091293)
Supplement: Supplementary file 1 [file biomolecules-15-01293-s001.zip › biomolecules-3737801-supplementary-fileS1_revised_3rd.pdf]

Supplementary File S1. Identification of a contig derived from the W chromosome in Okinawa population, and test for conservation of a W specific sequence between the two populations.

1. Methods

We conducted PCR to test the sex specificity of a specific sequence in contig 2879. We designed three primers, one forward primer and two reverse primers across the boundaries between the specific and common regions (Supplementary Figure 1a). The forward primer and one of the two reverse primers were designed on the common parts between contigs 1556 and 2879: Gho_ZW_Fw (5'-AGT AGA TGA TGA GAA AGA CCT CAG C-3') and Gho_ZW_Rv (5'-CCA AGG AAC ACG AAG AAA TAA CAC A-3'). The other reverse primer was designed on the specific part of contig 2879: Gho_W_Rv (5'-AAC ATC TAA AGT AAC CAA AGC TGC C-3'). Predicted sizes of amplicons were 181 bp and 283 bp for primer pairs Gho_ZW_F and Gho_ZW_R, and Gho_ZW_F and Gho_W_R, respectively. The genomic DNAs of three males and three females from each of the two populations, Okinawa and Ishigaki populations, were used as a template DNA. The PCR conditions were as follows: an initial denaturation at 94 °C for 2 min, followed by 30 cycles of 98 °C for 5 s, 60 °C for 15 s and 68 °C for 15 s, and finally 68 °C for 5 min for a final extension.

2. Results and Discussion

Whereas the 180 bp PCR amplicons by Gho_ZW_Fw and Gho_ZW_Rv were detected in all individuals used, the 280 bp PCR amplicons by Gho_ZW_Fw and Gho_W_Rv were observed only in females of the Okinawa population (Supplementary Figure 1b). This

suggests that contig 2879 was derived from a female-specific genomic region, i.e., a part of the W chromosomes of the Okinawa population. This W-specific region was not detected in the female genomic DNAs of the Ishigaki population; therefore, this region probably occurs only in the Okinawa population.

In theory, the primer pair of Gho_ZW_Fw and Gho_ZW_Rv would produce a longer amplicon with 3,175 bp predicted length in the females of the Okinawa population. However, the bands with a predicted size were not observed. This was probably due to the preferential amplification for shorter DNA fragments.
